# Rheumatic Immuno-related Adverse Events following Immunotherapy with Checkpoint Inhibitors: Adverse Drug Reaction, or Other?

**DOI:** 10.31138/mjr.32.2.186

**Published:** 2021-06-30

**Authors:** Ciro Manzo

**Affiliations:** Azienda Sanitaria Locale Napoli 3 sud, Internal and Geriatric Medicine Department – Geronthorheumatological Outpatient Clinic, Poliambulatorio “Mariano Lauro”, Sant’Agnello, Naples, Italy

Dear Editors,

I read with interest the case report entitled “Arthritis and myositis in a patient with programmed cell death-1 (PD-1) inhibitor pembrolizumab for lung cancer”, recently published in the Mediterranean Journal of Rheumatology.^1^

Since 2011, when the Food and Drug Administration (FDA) approved the use of ipilimumab - a fully human monoclonal antibody against cytotoxic-T-lymphocyte antigen-4 (CTLA4) - for patients with metastatic melanoma, the immunotherapy with checkpoint inhibitors has been recommended for an increasing variety of cancers, both in metastatic and adjuvant settings.

It is a common knowledge that the same mechanism of action of these immune check-point inhibitors (ICIs) can favor immune-related adverse events (IRAEs) which can affect multiple organ systems, and this risk is higher when two ICIs are used in combination. Triggered by the growing use of ICIs, an increasingly wide range of rheumatologic IRAEs have been described: arthritis, myositis, Sjogren’s syndrome, polymyalgia rheumatica (PMR) or PMR-like syndromes, among these.^2–4^

How were these rheumatologic IRAEs evaluated as an adverse drug reaction (ADR)? According to our recent systematic review regarding PMR and PMR-like syndromes,^5^ in most cases, only clinical judgment was used. Results were not very different when other different rheumatologic IRAEs were evaluated.^6–8^ This is a relevant discussion point, because within the field of pharmacovigilance, clinical judgment is often inferior to more structured methods of decision making that use simple algorithms.^9^ A number of algorithms have been proposed and some of these have been validated, but none of these have yet been accepted as gold standard. One commonly used algorithm is the ADR Probability Scale developed in 1981 by Naranjo and colleagues to standardize causality assessments.^10^ This scale classifies the probability that an adverse event is related to drug therapy. Naranjo’s scale is based on a list of 10 weighted questions, which examine factors such as the temporal association of drug administration and event occurrence, alternative causes if any, drug levels, and previous patient experience with the same drug. The key advantages of this scale are its simplicity of use and clarity, and a significant increase in inter- and intra-rater agreement compared with standard clinical examination alone. The sum of the scores ranges from −4 to +13, and is interpreted to reflect the strength of the probability that a drug has caused an ADR rather than the complication being a manifestation of the disease. A score > 9 is empirically defined as “definitely” having caused the ADR; a score between 5 and 8 indicates that the drug “probably” caused the ADR; scores between 1 and 4 indicate that the ADR was “possibly” caused by the drug; and a score < 1 indicate a “doubtful” association with the drug.

Recently, an EULAR task force confirmed that using this scale may help to assess the casual link between a specific rheumatic feature and ICIs therapy.^11^ However, when, using reported data, we were able to apply Naranjo’s scale to patients diagnosed with PMR following ICIs therapy, their total scores were almost never >4. Moreover, a significant number of the patients improved without ICI discontinuation, and there was no relapse when ICI was re-introduced.^5^

In the initially mentioned case report, arthritis and myositis appeared 5 and half months after pembrolizumab initiation.^1^ In general, rheumatologic IRAEs can appear several months after immunotherapy initiation. This is due in part to ICIs pharmacodynamic characteristics.

Are rheumatic IRAEs following ICIs therapy ADRs, or other?

It is common knowledge that arthritis, myositis, or other rheumatic inflammatory manifestations can be paraneo-plastic findings. In cancer patients on ICIs, this possibility should carefully be assessed and excluded through a follow-up. A few researchers proposed that this follow-up should last no less than two years.^12^ Besides, some rheumatic manifestations could be a coincidence, and not induced by ICIs therapy. For instance, an anti-citrullinated peptides antibodies (ACPA)-positive arthritis was reported in cancer patients following ICIs therapy.^13^ Also, patients’ response to therapy is unpredictable: in some cases, ICIs discontinuation was necessary, but when ICIs were reintroduced, rheumatologic IRAEs rarely reappeared.^14^

In conclusion, is the methodological approach of deductive type we found in most published literature the appropriate one? Are we going in the right direction?
